# Tryptophan as a Probe to Study the Anticancer Mechanism of Action and Specificity of α-Helical Anticancer Peptides

**DOI:** 10.3390/molecules190812224

**Published:** 2014-08-13

**Authors:** Guirong Li, Yibing Huang, Qi Feng, Yuxin Chen

**Affiliations:** 1 Key Laboratory for Molecular Enzymology and Engineering of the Ministry of Education, Jilin University, Changchun 130012, China; 2 School of Life Sciences, Jilin University, Changchun 130012, China; 3 National Engineering Laboratory for AIDS Vaccine, Jilin University, Changchun 130012, China

**Keywords:** anticancer peptides, mechanism of action, specificity, tryptophan

## Abstract

In the present study, a single tryptophan, as a fluorescence probe, was shifted from the N-terminus to the middle and to the C-terminus of a 26-residue α-helical anticancer peptide sequence to study the mechanism of action and specificity. The hydrophobicity of peptides, as well as peptide helicity and self-associating ability, were slightly influenced by the position change of tryptophan in the peptide sequence, while the hemolytic activity and anticancer activity of the peptide analogs remained the same. The tryptophan fluorescence experiment demonstrated that peptide analogs were more selective against LUVs mimicking cancer cell membranes than LUVs mimicking normal cell membranes. During the interaction with target membranes, the N-terminus of an anticancer peptide may be inserted vertically or tilted into the hydrophobic components of the phospholipid bilayer first. The thermodynamic parameters of the peptides PNW and PCW, when interacting with zwitterionic DMPC or negatively charged DMPS, were determined by ITC. DSC experiments showed that peptide analogs significantly altered the phase transition profiles of DMPC, but did not dramatically modify the phase transition of DMPS. It is demonstrated that hydrophobic interactions are the main driving force for peptides interacting with normal cell membranes, whilst, electrostatic interactions dominate the interactions between peptides and cancer cell membranes. Utilizing tryptophan as a fluorescence probe molecule appears to be a practicable approach to determine the interaction of peptides with phospholipid bilayers.

## 1. Introduction

A number of anticancer agents have been developed to treat cancer. Most chemotherapeutic agents have little or no selectivity against normal mammalian cells and consequently cause severe side effects [1]. In addition, cancer cells also develop resistance, for example, pumping out chemotherapeutic agents using multidrug resistance proteins [2]. Hence, the development of new classes of anticancer drugs has become critical. 

In recent years, membrane-active peptides have been widely studied due to their specific mode of action, which plays important roles in the host innate defense mechanism of many plants, insects and mammals [3,4]. Membrane-active peptides not only possess antibacterial or antifungal activities, but also possess anticancer activities [5,6]. Cationic anticancer peptides show strong selectivity to kill cancer cells compared to normal eukaryotic cells, due to the specific characteristics of the outer membrane of cancer cells, which contain more negative charges owing to the presence of negatively charged phosphatidylserine (PS) (3%–9% of the total membrane phospholipids) [7] and *O*-glycosylated mucines [8] than normal cells. The electrostatic interactions between anticancer peptides and negatively charged cancer cell membrane are favored [9]. In contrast, the electrostatic interactions between cationic anticancer peptides and normal eukaryotic cell membrane are weak due to the low overall charges of normal cells which contain natural zwitterionic phospholipid, e.g., sphingomyelin, phosphatidyl-ethanolamine and phosphatidylcholine [10]. Furthermore, the relatively higher number of microvilli on cancer cells compared with normal cells increases the surface area of tumor cell membrane and enables the binding of cationic peptides. Thus, due to the specific mode of action of cationic anticancer peptides on the cytoplasmic membrane, it is difficult to develop resistance of cancer cells since this would require substantial changes in the lipid composition of cancer cell membrane.

Although the mechanism of action of anticancer peptides has been well studied, there is no general consensus about it. A number of structure/activity studies have identified that certain parameters of cationic anticancer peptides, such as hydrophobicity, net charge, amphipathicity, secondary structure and oligomerization ability, have critical effects on the biological activities [6,11,12,13]. In a previous study, we have proved that the mechanism of action of cationic anticancer peptides against various cancer cells was necrosis resulting in fast cell membrane lysis, and hydrophobicity played a crucial role in the action [13]. 

Tryptophanes in peptide sequences have been well utilized as probes to detect membrane-active peptide structure and dynamics. It is reported that the spatial position and arrangement of tryptophanes affect membrane-active peptide adsorption and activity [14,15] and tryptophanes have been observed to modulate hydrophobic mismatches to maintain peptide stability and activity in lipid bilayer membranes [16]. The sensitivity of tryptophan emission to the polarity of the environment makes tryptophan fluorescence an important tool in studies of peptide structure and dynamics [17]. In this study, we used tryptophan on the hydrophobic face of an α-helical anticancer peptide as a probe to further illustrate the anticancer mechanism of action and specificity of cationic anticancer peptides. 

## 2. Results

### 2.1. Peptide Design

In previous studies, a 26-residue amphaipathic α-helical anticancer peptide A12L/A20L was obtained by replacing alanine with leucine at positions 12 and 20, respectively, on the hydrophobic face of peptide V13K, an analog of peptide V681 [18,19,20]. We have systematically studied the effects of hydrophobicity and helicity of α-helical cationic anticancer peptides on the anticancer mechanism of action [12,13]. In this study, peptide A12L/A20L referred to as parent peptide P was used as a framework to shift a single tryptophan residue from position 2 in the parent sequence to position 12 and position 24, substituting for the original leucine and isoleucine, respectively. We replaced leucine or isoleucine in position 2 after the corresponding tryptophan shifts, maintaining the equal overall amino acid composition and hydrophobicity of the peptides. Positions 2, 12 and 24 in the sequence are close to the N-terminus, the middle and the C-terminus, respectively, where we utilized the tryptophan residue as a probe molecule to study the mechanism of anticancer action and the specificity of α-helical anticancer peptides. Thus, the three peptides were named peptide with N-terminal tryptophan (peptide P, PNW), peptide with middle-position tryptophan (PMW) and peptide with C-terminal tryptophan (PCW) based on the corresponding position of the tryptophan substitution, as shown in Table 1. 

**Table 1:** Sequence of peptides used in this study.

Peptides	Sequence ^a^
P(PNW)	Ac-K-**W**-K-S-F-L-K-T-F-K-S-L-K-K-T-V-L-H-T-L-L-K-A-I-S-S-amide
PMW	Ac-K-I-K-S-F-L-K-T-F-K-S-**W**-K-K-T-V-L-H-T-L-L-K-A-I-S-S-amide
PCW	Ac-K-I-K-S-F-L-K-T-F-K-S-L-K-K-T-V-L-H-T-L-L-K-A-**W**-S-S-amide

^a^ One-letter codes are used for amino acid residues; all amino acids are l-amino acids. Bold letters show the positions of tryptophan in the sequence.

A control peptide (peptide C, Ac-ELEKGGLEGEKGGKELEK-amide) designed to exhibit negligible secondary structure,* i.e.*, a random coil, in benign and in the presence of 50% TFE was employed as a standard peptide for temperature profiling during RP-HPLC to monitor peptide dimerization ability of peptide, as shown in the previous study [21].

### 2.2. Peptide Secondary Structure

**Figure 1:**
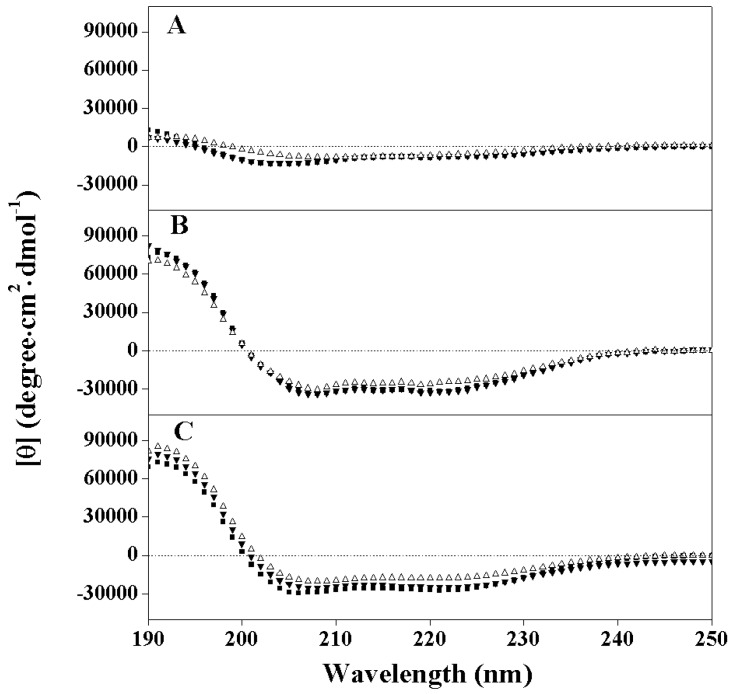
Circular dichroism (CD) spectra of the peptide analogs. Panel A denotes the CD spectra of peptide analogs in mild KP buffer, while Panel B denotes the CD spectra obtained in the presence of 50% 2,2,2-trifluoroethanol. Panel C denotes the CD spectra of peptide analogs in mild KP buffer with 10mM SDS. The symbols used are as follows: ■ for peptide PNW, ▼ for peptide PMW, Δ for peptide PCW.

Peptide secondary structure was measured by CD spectroscopy on a Jasco-810 spectropolarimeter under mild buffer conditions (50 mM KH_2_PO_4_/K_2_HPO_4_, and 100 mM KCl, pH 7), in 50% TFE and under mild buffer conditions with 10 mM SDS to mimic the hydrophobic environment of the cell membrane (Figure 1 and Table 2). The molar ellipticity values in the different environments are shown in Table 2. All peptide analogs show negligible helical structures with molar ellipticity values ranging from −5900 to −7800 under mild conditions. In contrast, all peptide analogs were induced into highly helical structures with molar ellipticity values ranging from −24,300 to −31,700 in the hydrophobic environment of 50% TFE and molar ellipticity values ranging from −17,800 to −27,300 in the hydrophobic buffer environment in the presence of 10 mM SDS. Compared to the peptide PCW, peptides PNW and PMW showed comparatively more helical structure in mild buffer, in the presence of 50% TFE and in the prescence of 10 mM SDS. It is obvious that the tryptophan residue played an important role in stabilizing the helical structure at the N-terminus or in the middle position of the peptide sequence. 

### 2.3. Hydrophobicity and Peptide Self-Association 

The relative hydrophobicity of the peptides was determined by measuring their RP-HPLC retention times at 25 °C. The retention times of peptides are highly sensitive to the helicity of peptides upon the interaction between the peptide with the hydrophobic environment of the column matrix [13,19,22]. From Table 2, the relative hydrophobicity of peptide analogs is in the order PCW < PMW < PNW. Although the amino acid compositions of three peptide analogs are exactly the same, it is interesting to see that PNW and PMW showed stronger relative hydrophobicity than PCW, which is consistent with the CD results indicating that PNW and PMW are more helical than PCW in the hydrophobic environment. It is well known that the chromatography conditions characteristic of RP-HPLC (hydrophobic stationary phase, non-polar eluting solvent) induce helical structures in potentially helical peptides [18,22] in a manner similar to that of the helix-inducing solvent trifloroethanol (TFE). Peptides which are induced into an amphipathic á-helix by interacting with a hydrophobic RP-HPLC stationary phase exhibit preferred binding of their non-polar face with the stationary phase. Indeed, Zhou* et al.* [23] clearly demonstrated that, due to this preferred binding domain, amphipathic á-helical peptides are considerably more retentive than non-amphipathic peptides of the same amino acid composition. Hence, peptides PNW and PMW showed stronger helical structure in the hydrophobic environment, thus exhibited stronger retentive behaviors than PCW.

**Table 2:** Biophysical data of the peptide analogs.

Peptides	*t*_R_^a^	Benign ^b^		50%TFE ^c^		SDS in Buffer ^d^		*P*_A_ (min) ^e^
		[θ]_222_	% Helix ^f^	[θ]_222_	% Helix	[θ]_222_	% Helix	
PNW	46.8	−7800	24	−31700	99	−27,300	86	5.7
PMW	46.5	−7400	23	−31900	100	−25,050	79	5.3
PCW	45.6	−5900	18	−24300	76	−17,800	56	4.5

^a^ Denotes the retention time at 25 °C by RP-HPLC; ^b^ The mean residue molar ellipticities, [θ]_222_ (degree cm^2^ dmol^−1^) at wavelength 222 nm were measured at 25 °C in KP buffer (100 mM KCl, 50 mM KH_2_PO_4_/K_2_HPO_4_, pH 7.4); ^c^ The mean residue molar ellipticities, [θ]_222_ (degree.cm^2^.dmol^−1^)at wavelength 222 nm were measured at 25 °C in KP buffer with 50% TFE; ^d^ The mean residue molar ellipticities, [θ]_222_ (degree cm^2^ dmol^−1^)at wavelength 222 nm were measured at 25 °C in KP buffer with 10 mM SDS; ^e^*P*A denotes the association parameter of each peptide during the RP-HPLC temperature profiling, which is the maximal retention time difference of ((*t*_R_^t^ − *t*_R_^5^ for peptide analogs) − (*t*_R_^t^− *t*_R_^5^ for control peptide C)) within the temperature range, and (*t*_R_^t^ − *t*_R_^5^) is the retention time difference of a peptide at a specific temperature (*t*) compared with that at 5 °C; ^f^ The helical content (in percentage) of a peptide relative to the molar ellipticity value of peptide PMW in 50% TFE.

**Figure 2:**
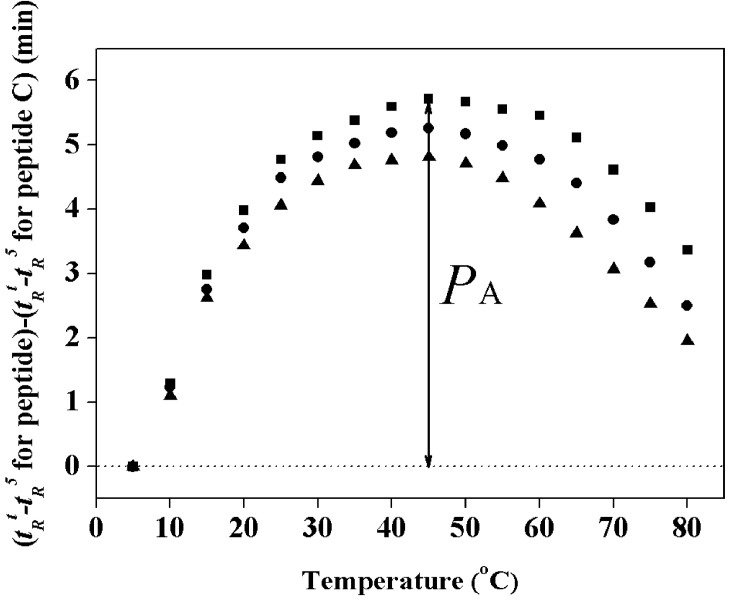
Peptide self-association ability during RP-HPLC temperature profiling. The retention behavior of the peptides was normalized to that of the random coil peptide C through the expression (*t*_R_^t^ −* t*_R_^5^ for peptide analogs) minus (*t*_R_^t^ −* t*_R_^5^ for control peptide C), where *t*_R_^t^ is the retention time at a specific temperature of the anticancer peptides or the random coil peptide, and *t*_R_^5^ is the retention time at 5 °C. Symbols used are: ■ for peptide PNW; ● for peptide PMW; ▲ for peptide PCW.

We utilized the RP-HPLC temperature profiling technique to determine the peptide self-association ability in aqueous solution which we believe is a very important parameter to understand anticancer activity [24]. When the self-association ability of a peptide is too strong in aqueous solution, it could decrease the ability of the peptide to dissociate and penetrate into membranes to kill target cells. Figure 2 shows the RP-HPLC temperature profiling of peptide analogs during RP-HPLC from 5 to 80 °C. The curves were normalized with the control peptide C which is a monomeric random coil peptide in both aqueous and hydrophobic media, showing only the general temperature effects [21]. The peptide self-association parameter (*P*_A_) is the maximum change in peptide retention time relative to the control peptide C, to quantify the self-association ability of peptides in solution. The details of how to determine peptide self-association parameter were reported previously [18]. From Table 2, it is clear that the ability of peptides to self-associate has the same order as the hydrophobicity of the peptide analogs,* i.e.*, PCW < PMW < PNW. It is thus indicated that the self-association ability of cationic α-helical peptides in an aqueous environment is correlated with hydrophobicity and helicity of the peptides, which is consistent with the previous work [19].

### 2.4. Anticancer Activity and Hemolytic Activity 

In the previous study, the parent peptide PNW showed broad-spectrum anticancer activities against a variety of cancer cell lines [13]. Thus, in this study the HeLa cell line was selected as an example to evaluate the anticancer activity of the peptide analogs. The IC_50_ values for HeLa cells were collected to provide an evaluation of anticancer activity. As shown in Table 3, there was no significant difference in anticancer activity among peptide analogs. This indicated that the position of the tryptophan probe molecule in peptide sequence had no obvious effect on the anticancer activity against HeLa cells. 

The hemolytic activity of peptide against human red blood cells was determined to evaluate the toxicity toward normal eukaryotic cells. All peptide analogs had the same hemolytic activity (Table 3). Like in the anticancer activity, the position of the tryptophan probe molecule in peptide sequence had no effect on hemolytic activity against human red blood cells.

**Table 3:** Anticancer activity (IC50) and hemolytic activity (MHC) of peptide analogs against HeLa cells and human red blood cells.

Peptides	IC_50_ (μM) ^a^	MHC (μM) ^b^
PNW	2.35 ± 0.28	10.41 ± 0.02
PMW	2.47 ± 0.18	10.41 ± 0.01
PCW	2.33 ± 0.12	10.41 ± 0.01

^a^ Anticancer activity (IC_50_) represents the concentration of peptides at which cell viability was reduced by 50% in comparison to untreated cells. ^b^ Hemolytic activity (minimal hemolytic concentration) was determined on human red blood cells after incubating with peptides for 1 h (hRBC).

### 2.5. Tryptophan Fluorescence and Quenching Experiments 

The fluorescence emission of the tryptophan residue was used to monitor the binding of peptides to liposomes, since the fluorescence of the tryptophan residue is sensitive to different environments. The fluorescence emission maxima of peptides exhibited a blue shift and a marked increase in emission intensity when the peptides with tryptophan residues inserted into a hydrophobic environment, such as the hydrophobic core of the cytoplasmic membrane [25]. In order to further investigate the specificity of peptide analogs in different membrane-mimicking environments, the phospholipid compositions used in this study were PC/SM/PE/cholesterol (4.5:4.5:1:1 w/w) mimicking the composition of normal mammalian cell membrane and PC/SM/PE/PS/cholesterol (4.35:4.35:1:0.3:1 w/w) mimicking the composition of cancer cell membranes [9]. These LUV model membrane systems were named as normal LUVs and cancer LUVs, respectively. 

**Figure 3:**
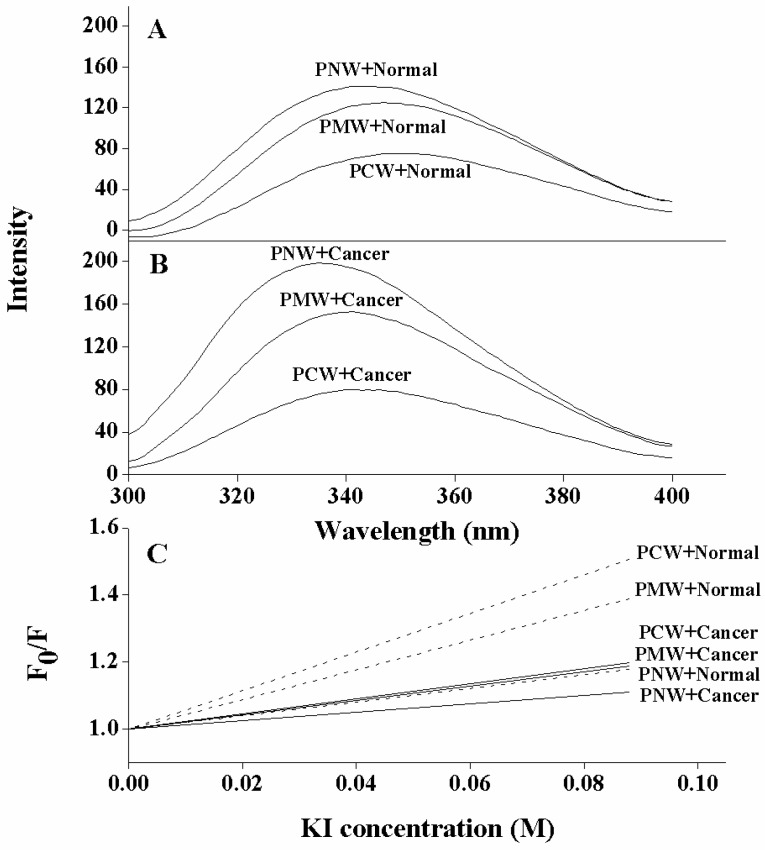
Tryptophan fluorescence and quenching of peptides at pH 7.4, 25 °C. Panel A and Panel B show spectra in LUVs mimicking membranes of normal cells (PC/SM/PE/cholesterol (4.5:4.5:1:1 w/w)) and cancer cells (PC/SM/PE/PS/cholesterol (4.35:4.35:1:0.3:1 w/w)) at a peptide to lipid ratio of 1:50, respectively. Panel C shows the Stern-Volmer plots of KI quenching. “Normal” denotes LUVs mimicking normal membrane; “Cancer” denotes LUVs mimicking cancer membrane.

Comparing the fluorescence emission maxima of the three peptides in both model membrane systems (cancer LUVs and normal LUVs), the PNW peptide showed the largest blue shifts and the most increases in emission intensity than other two peptides, either interacting with cancer LUVs or normal LUVs; whereas PCW peptide showed the smallest blue shifts and the least increases in emission intensity among the three (Figure 3A,B and Table 4). It is clear that the tryptophan residue on PNW relocated into the deep hydrophobic environment when interacting with membranes; in contrast, the tryptophan on PCW inserted much shallower into the hydrophobic membrane, and PCW showed a particularly negligible interaction with normal LUVs. Thus, for peptide P, activity is position-dependent when interacting with membranes, that is, the N-terminus of peptide inserted much deeper into phospholipid bilayer membrane than the C-terminus. More importantly, compared to the interaction with normal LUVs, peptides inserted much deeper into cancer LUVs, exhibiting larger increases on values of blue shifts and fluorescence intensity, respectively.

**Table 4:** Fluorescence intensity changed and blue shifts in emission wavelength maxima of Trp fluorescence upon exposure of peptides to freshly prepared vesicles.

Peptides	Membranes ^a^	Intensity change ^b^	Blue shift (nm) ^c^
PNW	cancer LUVs	81	14
	normal LUVs	23	5
PMW	cancer LUVs	38	9
	normal LUVs	11	3
PCW	cancer LUVs	2	6
	normal LUVs	0	0

^a^ Cancer LUVs denote cancer-mimicking membrane and normal LUVs denote model membrane mimicking normal cells. See Experimental for details; ^b^ Fluorescence intensity change is the difference of a peptide at maximal Trp fluorescence intensity in liposome compared with that in Hepes buffer. The values were generally reproducible within ±1 nm; ^c^ Blue shift in emission wavelength maxima is the difference of a peptide in liposome compared with that in HEPES buffer. The values were generally reproducible within ±0.5 nm.

The tryptophan fluorescence intensity was decreased in a concentration-dependent manner by the addition of the water soluble quencher KI. The Stern-Volmer plots (Figure 3C), where the accessibility of tryptophan to aqueous quencher was plotted, showed that the fluorescence of peptide PCW was quenched to the greatest degree compared to that of PMW and PNW, indicating that PCW inserted the most shallow than other two peptides into membranes, since peptide PNW possessed the lowest values of slope in either cancer LUVs or normal LUVs whereas peptide PCW possessed the highest values of slop in both membrane systems. 

Again, it further proved that the N-terminus of peptide inserted deeper than the C-terminus into hydrophobic membrane environments. Meanwhile, it also showed that all peptide analogs were more selective toward the negatively-charged cancer-mimicking model membrane than a zwitterionic normal model membrane.

### 2.6. Isothermal Titration Calorimetry (ITC)

**Figure 4:**
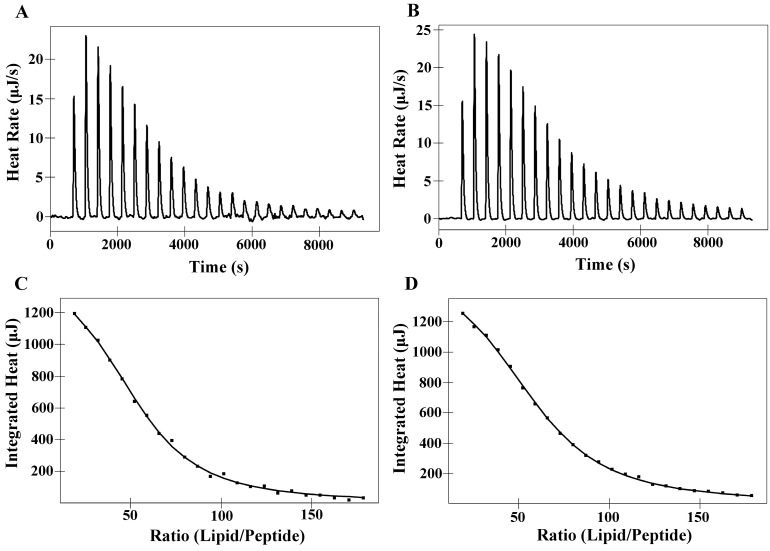
Isothermal titration of PNW and PCW with DMPC LUVs at 25 °C. (**A**) DMPC LUVs titration into PNW; (**B**) DMPC LUVs titration into PCW. Panels (**C**) and (**D**) denote the heat of reaction of peptides PNW and PCW (measured by peak integration) as a function of lipid/peptide molar ratio, respectively. The solid line denoted the best fits to experimental data.

The interaction of the peptides PNW and PCW with phospholipids was investigated using ITC at 25 °C, in which DMPC or DMPS LUVs were injected into an ITC cell containing peptide solutions of PNW or PCW. Based on the tryptophan fluorescence and quenching experiments, PNW and PCW could cover the trend of peptide-membrane interaction, thus, PMW was not included in the liposome experiments. The interaction of peptides with phospholipid bilayer membrane is entropy-driven [26]. Figure 4 shows the thermodynamic profiles of PNW or PCW binding to DMPC LUVs and corresponding thermodynamic parameters are presented in Table 5. It is clear to see that the endothermic heat flow decreased with the increase of the number of injections and the free peptide concentration in the cell decreased simultaneously. The endothermic binding reaction of peptide PNW ceased after 20 injections, when all of peptide in bulk bound to DMPC LUVs (Figure 4A) and the further addition of DMPC LUVs caused dilution heat. However, the endothermic binding reaction of PCW ceased after 22 injections. It is indicated that peptide PNW was more susceptible to DMPC LUVs than PCW. The affinity constant *K*a value of peptide PNW (5.00 × 10^3^ M^−1^) to DMPC LUVs were higher than that of peptide PCW (4.15 × 10^3^ M^−1^) (Table 5). It is indicated that peptide PNW bound stronger to DMPC compared to peptide PCW. 

**Table 5:** Thermodynamic parameter of peptide binding to DMPC and DMPS LUVs.

Membranes	Peptides	*K*a ^a^ [M]^−1^× 10^3^	∆*H *^b ^(kJ M^−1^)	∆*G *^c ^(kJ M^−1^)	T ^d^ ∆*S *(kJ M^−1^)	∆*S *^e^(J M^−1^K^−1^)	N ^f^
DMPC	PNW	5.00	10.36	−21.12	31.48	105.60	52.51
	PCW	4.15	10.93	−20.64	31.57	105.90	58.60
DMPS	PNW	20.69	−4.74	−24.64	19.90	66.72	16.91
	PCW	17.70	−3.20	−24.24	21.04	70.58	18.96

^a^ Affinity constant directly obtained in the ITC experiments at 25 °C. ^b^ Total binding enthalpy directly obtained in the ITC experiments at 25 °C. ^c^ Free energy of binding, Δ*G* = Δ*H *− TΔ*S.* ^d^ Temperature, the ITC experiments were tested at 298.15 K. ^e^ Entropy of binding directly obtained in the ITC experiments. ^f^ Binding stoichiometry.

**Figure 5:**
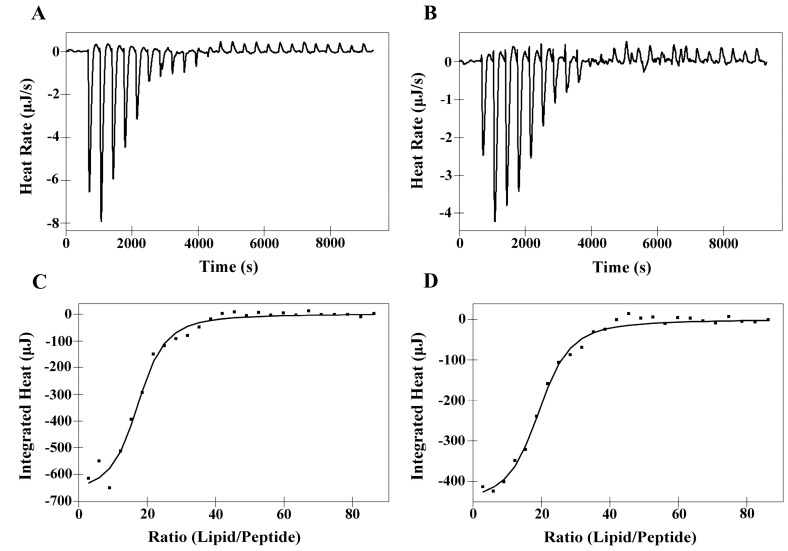
Isothermal titration of PNW and PCW with DMPS LUVs at 25 °C. (**A**) DMPS LUVs titration into PNW; (**B**) DMPS LUVs titration into PCW. Panels (**C**) and (**D**) denote the heat of reaction of peptides PNW and PCW (measured by peak integration) as a function of lipid/peptide molar ratio, respectively. The solid line denoted the best fits to experimental data.

Figure 5 illustrates titration experiments where DMPS LUVs were injected into an ITC cell containing PNW or PCW solution, which was an exothermal and entropy-driven process. ∆*G* values of both peptides binding to DMPS LUVs remained relatively constant (−24.24 to −24.64 kJ M^−1^) and the affinity constant *K*a of peptide PNW was slightly higher than that of peptide PCW, which indicating that the degrees of binding of peptide PNW to DMPS were stronger compared to peptide PCW to DMPS (Table 5). However, as shown in Table 5, the *K*a and ∆*G* values of peptides to DMPS were significantly larger than that of peptides to DMPC. It is indicated that the interaction of peptides with DMPS is stronger than peptides with DMPC due to electrostatic interaction.

### 2.7. Differential Scanning Calorimetry Experiment (DSC)

**Figure 6:**
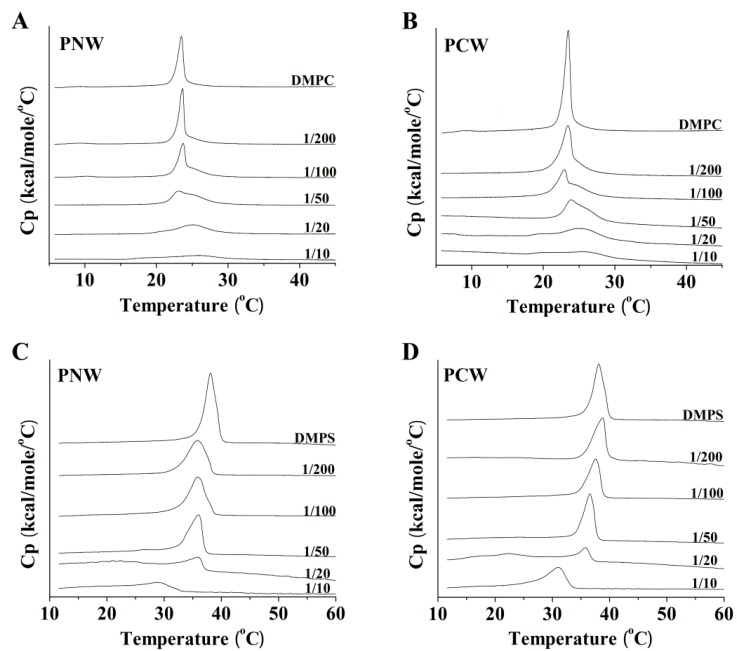
Effect of peptide concentration of the gel-liquid crystalline phase transition of DMPC MLVs and DMPS MLVs. DSC thermograms are shown for the effect of increasing concentration of peptides PNW and PMW on the thermotropic phase behavior of DMPC MLVs and DMPS MLVs. The control of pure lipid alone is shown at the top. The peptide/lipid molar ratios are indicated.

DSC heating thermograms illustrated the effects of peptides PNW and PCW on the thermotropic phase behavior of DMPC MLVs or DMPS MLVs (Figure 6). Dimyristoyl (DM) lipids were chosen for the calorimetric measurements because the phase transition temperatures of these lipids occur at room temperature which facilitates handling and study of their phase transition by DSC. As shown in Figure 6A,B, in the absence of peptides, DMPC MLVs exhibited two endothermic events on heating, a weakly energetic pretransition near 10 °C and a strongly energetic main phase transition near 23 °C. The two peptides incorporated into DMPC MLVs had great effect on the thermotropic phase behavior, increasing the temperature and reducing the coorperativity of the main phase transition at higher peptide concentrations and abolishing pretransition at lower peptide concentrations. Abolishment of the pretransition of DMPC implies that the two peptides interacted with headgroups of phospholipids. Increases in the temperature of the main phase transition suggest the reduction of DMPC membrane fluidity, and the broadening of the peaks implies the peptides interacting with hydrocarbon chain of DMPC. As shown in Figure 6C,D, the presence of two peptides in DMPS MLVs slightly decreased the temperature of the main phase transition until the ratio of peptide/lipid reaching 1:50. The incorporation of large amounts of peptides into DMPS MLVs (peptide:DMPS = 1:20) significantly altered their thermotropic phase behaviors. It seems that the two peptides interacted with DMPS through electrostatic interaction at lower concentrations; in contrast, at higher concentrations, peptides interacted strongly with phospholipid bilayer membrane with additional force other than electrostatic interaction. It is interesting to see that the increase of peptide concentrations showed different influences on the changes of transition temperature of DMPC MLVs and DMPS MLVs. For DMPC MLVs, transition temperature seems to increase with the increase of peptide concentration; whilst, for DMPS MLVs, transition temperature seems to decrease with the increase of peptide concentration. As illustrated in the previous study [27], the peak of the main phase transition of DMPC is complicated with increasing peptide concentrations. It may be attributed to the fact that peptide-poor and peptide-rich phospholipid domains can be formed in DMPC phospholipid bilayer. Moreover, phase transition temperatures of peptide-poor and peptide-rich phospholipid have different variation while changing concentration of peptides.

## 3. Discussion

In the present study, in order to investigate the mechanism of anticancer action and specificity of α-helical anticancer peptides, the tryptophan on the parent peptide was utilized as a fluorescence probe by shifting it from the N-terminus to the middle and the C-terminus of the parent peptide sequence, respectively, without changing peptide amino acid composition. As shown in Table 2, Figure 1 and Figure 2, it is clear that the tryptophan position changes on an α-helical peptide exhibited the similar trend of effects on hydrophobicity as well as on peptide secondary structure and self-associating ability. This can be attributed to the fact that peptides with stronger helicity in solution usually exhibit more complete non-polar faces or polar faces, thus showing higher relative hydrophobicity as well as stronger self-association by binding the non-polar faces of two peptide molecules together or to the stationary phase during RP-HPLC [19]. The position changes of tryptophan residue in peptide sequence have no significant influence on hemolytic activity and anticancer activity, which shows that the anticancer and membrane disruption activities of α-helical anticancer peptides are not sequence-dependent. Thus, there is no receptor on the membrane for these membrane-active peptides which disrupt lipid bilayer membrane in a necrotic way [13].

Several mechanisms of action of membrane-active peptides have been proposed, including “barrel-stave” mechanism, “carpet” mechanism, “toroidal pore” mechanism and “disordered toroidal pore” mechanism [28]. In the previous study, we proposed “membrane discrimination” mechanism of membrane-active peptide whose sole target is the biomembrane [18,19,29], based on a “barrel-stave” mechanism [30] in eukaryotic cells and a “carpet” mechanism [31] in prokaryotic cells. In eukaryotic cells, bundles of amphipathic α-helices form transmembrane channels/pores, as their hydrophobic surfaces interact with the lipid core of the membrane and the hydrophilic surfaces point inward, producing an aqueous pore. 

In this study, the anticancer peptides may use an approach of peptide insertion into membrane as described in the “membrane discrimination” mechanism. In tryptophan fluorescence experiments (Table 4 and Figure 3A,B), while peptide analogs interacted with cancer LUVs, the tryptophan residue inserted into the hydrophobic lipid of the bilayer and the order of tryptophan insertion depth of peptide analogs in cancer LUVs is PCW < PMW < PNW. Whilst interacting with normal LUVs, peptides exhibited the same tendency as with cancer LUVs, that is PCW < PMW < PNW. Based on the fluorescence and quenching data, the N-terminus of peptide P would insert much deeper into the phospholipid membrane than the C-terminus with a vertical or tilted position or maybe a comparatively parallel orientation but with Trp oriented to the inside of the membrane, which is consistent with the “membrane discrimination” mechanism, that is, in eukaryotic cells, bundles of amphipathic α-helices form transmembrane channels/pores. The quenching assays further proved that the tryptophan residues in all three peptides were inserted into the hydrophobic lipid of the bilayer with an order of insertion depth as PCW < PMW < PNW (Figure 3C). Moreover, it is also demonstrated that all peptide analogs were more selective to cancer-mimicking membrane attributing to the presence of anionic PS than the model membrane mimicking normal cells.

ITC data showed that the bindings of PNW and PCW to phospholipid bilayers were significantly influenced by the specific lipid composition of model membranes and the different type of forces (Table 5, Figure 4 and Figure 5). According to Ross,* et al.*, positive values of Δ*H* show that the main interaction between peptides and membrane is from hydrophobicity; in contrast, negative values of Δ*H* represent the major forces of van der Waals force and electrostatic interactions [32]. Hence, in this study, PNW and PCW exhibited selectivity between normal cells and cancer cells mainly due to the different interactions between peptides and membrane, that is, peptides show stronger interactions with cancer cells since there are not only hydrophobic interaction between peptides and membrane but also electrostatic interaction. However, the degrees of affinity of peptide PNW to zwitterionic DMPC membrane and anionic DMPS membrane were greater than peptide PCW (Figure 4 and Figure 5 and Table 5), which can be attributed to higher helicity, relative hydrophobicity and self-association ability of peptide PNW than those of peptide PCW. 

As shown in Figure 6, DSC results exhibited different effects of hydrophobic interaction and electrostatic interaction during the incorporation of the cationic anticancer peptides PNW and PCW on the thermotropic phase behavior of phospholipid MLVs. Compared to the incorporation of the two peptides into anionic DMPS MLVs, their incorporation into zwitterionic DMPC MLVs caused significantly decreases in the transition temperature, enthalpy and cooperativity of the main phase transition. It is implied that the two peptides inserted into the hydrophobic core of zwitterionic DMPC MLVs to disturb packing of phospholipid hydrocarbon chain; in contrast, for peptide interaction with anionic DMPS MLVs, both the electrostatic interaction and the hydrophobic interaction may affect the peptide entry into the membrane. For the interaction with DMPS MLVs, the peptides interacted with anionic DMPS MLVs by electrostatic interaction at low concentrations and the peptides inserted into hydrophobic core due to the hydrophobic interaction at high concentrations. These results are consistent with the previous studies that the electrostatic interaction and the hydrophobic interaction are the critical properties and the main driving forces in the mechanism of action and selectivity of membrane-active peptides [33]. 

In this study, it was interesting to see that the biophysical studies exhibited more sensitive results on the peptide selectivity of different membranes than biological data. This phenomenon may attribute to the limited substitutions on peptide sequence which can only be reflected by the subtle changes of relative hydrophobicity and secondary structure.

## 4. Experimental 

### 4.1. Reagents

Rink amide 4-methylbenzhydrylamine resin (MBHA resin; 0.8 mmol/g), all of the *N*-α-Fmoc protected amino acids and coupling reagents for peptide synthesis, and trifluoroacetic acid (TFA) were purchased from GL Biochem (Shanghai, China). Cholesterol, egg phosphatidylcholine (PC), porcine brain phosphatidylserine (PS), *E. coli.* phosphatidylethanolamine (PE), egg sphingomyelin (SM), 1,2-dimyristoyl-*sn*-glycero-3-phosphocholine (DMPC) and 1,2-dimyristoyl-*sn*-glycero-3-phospho-L-serine (sodium salt) (DMPS) were purchased from Avanti Polar Lipids, Inc. (Alabaster, AL, USA). Dichloromethane, *N,N*-dimethylformamide (DMF), and 2,2,2-trifluoroethanol (TFE) were purchased from JinTai Chemicals (Changchun, China). Acetonitrile (HPLC grade) was obtained from Fisher (Beijing, China). 

### 4.2. Peptide Synthesis and Purification

The peptides were synthesized by the solid-phase method using Fmoc (9-fluorenylmethoxycarbonyl) chemistry as described previously [18]. The crude peptides were purified on a LC-6A preparative reversed-phase high performance liquid chromatograph (RP-HPLC, Shimadzu, Kyoto, Japan) using a Zorbax 300 SB-C8 column (250 × 9.4 mm inner diameter, 6.5 μm particle size, 300 Å pore size; Agilent Technologies, Santa Clara, CA, USA) with a linear AB gradient at a flow rate of 2 mL/min. Mobile phase A was 0.1% aqueous trifluoroacetic acid (TFA) in water and B was 0.1% TFA in acetonitrile. The purity of peptides was verified by analytical RP-HPLC. The purified peptides were further characterized by mass spectrometry and amino acid analysis.

### 4.3. Analytical RP-HPLC and Temperature Profiling of Peptides

Peptide samples were analyzed on a Shimadzu LC-20A HPLC column. Runs were performed on a Zorbax 300 SB-C8 column (150 × 4.6 mm ID, 5 μm particle size, 300Å pore size) from Agilent Technologies, using a linear AB gradient (1% acetonitrile/min) and a flow rate of 1 mL/min, in which eluent A was 0.1% aqueous TFA in water and eluent B was 0.1% TFA in acetonitrile. Temperature profiling analyses during RP-HPLC were performed in 5 °C increments, from 5 °C to 80 °C, as described previously [21].

### 4.4. Circular Dichroism Spectroscopy

Circular dichroism (CD) spectra were acquired with a 0.02 cm path length quartz cuvette on a Jasco J-810 spectropolarimeter (Jasco, Easton, MD, USA) at 25 °C as described previously [12]. The concentration of 75 μM peptides was measured in benign buffer (50 mM KH_2_PO_4_/K_2_HPO_4_, and 100 mM KCl (pH 7.4)), benign buffer with 50% TFE and benign buffer with 10 mM SDS at 25 °C. The mean residue molar ellipticities were calculated by the equation [θ] = θ/10*l*C_M_n [18] and θ is the ellipticity in millidegrees, *l* is the optical path length of the cuvette in centimeters, C_M_ is the peptide concentration in mole/liter, and n is the number of residues in the peptide [13]. The values of mean residue molar ellipticities of the peptide analogs at 222 nm were used to determine the relative helicity of the peptides.

### 4.5. Measurement of Anticancer Activity

The MTT assay has been used to test cytotoxicity of reagents and cell viability. Human cervix carcinoma cells (HeLa cells) were seeded in 96-well plates and incubated with serially 2-fold diluted concentration of different peptides (μM) for 1 h at 37 °C. As a negative control, cells were cultured without addition of the peptides. Thereafter, 20 μL of 5 mg/mL MTT solution in PBS were added to cells and treated for 4 h at 37 °C. The formazan crystals were dissolved by adding 150 μL dimethyl sulfoxide (DMSO) just before spectrometric determination. The absorbance was determined at 490 nm. The results were expressed as IC_50_, representing the concentration at which cell ability was reduced by 50%. The cytotoxicity assays were repeated in triplicates.

### 4.6. Measurement of Hemolytic Activity

Peptide samples were serially diluted by PBS in 96-well plates (round bottom) to give a volume of 70 μL sample solution in each well. Human erythrocytes anticoagulated by EDTAK_2_ were collected by centrifugation (1000 rpm) for 5 min, and washed twice by PBS, then diluted to a concentration of 2% in PBS. 70 μL of 2% erythrocytes were added to each well to give a final concentration of 1% human erythrocytes in each well and plates were incubated at 37 °C for 1 h. The plates were then centrifuged for 10 min at 3,000 rpm and supernatant (90 μL) was transferred to a 96-well plate (flat bottom). The release of hemoglobin was determined by measuring the absorbance of the supernatant at 540 nm. The hemolytic activity was determined as the minimal peptide concentration that caused hemolysis (minimal hemolytic concentration, MHC). Erythrocytes in PBS and distilled water were used as control of 0% and 100% hemolysis, respectively.

### 4.7. Preparation of MLVs and LUVs 

To mimic cancer cell and normal cell membranes, desired phospholipid powders were mixtured under certain ratios. Phospholipid mixtures were dissolved in chloroform, dried by N_2_ flow, and then vacuumed overnight to remove the trace of organic solvents. The lipids were hydrated in 10 mM HEPES and 150 mM NaCl buffer (pH 7.4) and extensively vortexed above the phase-transition temperature of phospholipid, obtaining multilamellar large vesicles (MLVs). To obtain large unilamellar vesicles (LUVs), the MLVs was exposed to five freeze-thaw cycles and pass 21 times through two polycarbonate membrane (0.1 μm) with a mini-extruder above the phase-transition temperature of phospholipids. The phospholipid concentrations were determined by phosphorus analysis [34].

### 4.8. Tryptophan Fluorescence and Quenching Experiments

LUV liposomes to mimic cancer cell membrane (PC/SM/PE/PS/cholesterol = 4.35:4.35:1:0.3:1, w/w) and normal cell membrane (PC/SM/PE/cholesterol = 4.5:4.5:1:1, w/w) [9] were prepared. Each peptide (2 μM) was added to 1 mL of HEPES buffer (pH 7.4) containing 0.1 mM LUV liposomes and the peptide/liposome mixture was allowed to interact at room temperature for 10 min. The fluorescence intensity of tryptophan was detected by a Shimadzu RF-5301PC spectrofluorometer with an excitation wavelength of 280 nm and emission wavelength range from 300 to 400 nm. Slit widths of excitation and emission are both 5 nm. The fluorescence spectrum of each peptide with liposomes was subtracted from the spectrum of liposome alone. KI quenching experiments were carried out at an excitation wavelength of 280 nm. Small aliquots (10 μL) of KI were added from a 2 M stock solution to peptides in the absence or presence of LUV liposomes. The experimental data were plotted according to the Stern-Volmer equation F_0_/F = 1 + *K*sv[Q], where F_0_ and F are the fluorescence in the absence and presence of a quencher at concentration [Q], respectively, and *K*sv is the Stern-Volmer quenching constant [35].

### 4.9. Isothermal Titration Experiment (ITC)

The affinity of peptides with LUV liposomes were detected by a Nano ITC2G isothermal titration calorimeter (TA Instrument Corp., New Castle, DE, USA) at 25 °C, under HEPES buffer condition (10 mM HEPES, and 150 mM NaCl (pH 7.0)). To avoid air bubbles, peptides and LUV solutions were degassed under vacuum 500 mmHg, for 14 min before using. Titrations were performed by injecting aliquots of LUVs (lipid concentrations of DMPC and DMPS were 14.75 mM and 14.25 mM, respectively) into the calorimeter cell containing peptide solution (peptide concentrations varying between 25 and 50 μM) with 6 min waiting time between injections and 300 rpm of stirring rate. Data analysis was performed using the NanoAnalyze program provided by TA Instruments. Noises were controlled by the deduction of buffer signals in the presence or absence of liposomes. The vesicles sizes of peptide-exposed lipids are similar in DMPC and DMPS solutions; in contrast, the vesicles sizes of peptide-free lipids of DMPC and DMPS are similar to each other, but in comparatively smaller sizes than peptide-exposed lipids, respectively (data not shown).

### 4.10. Differential Scanning Calorimetry (DSC)

DSC experiments were performed on a MicroCal VP-DSC (Microcal Inc., Northampton, MA, USA). MLVs were prepared under Tris buffer (50 mM Tris-HCl, 100 mM NaCl, and 10 mM EDTA (pH 7.4)) at a temperature 10–15 °C above the phase transition of DMPC or DMPS. Lipid and peptide were mixed at the required molar ratios (peptide/lipid for 1/200, 1/100, 1/50, 1/25, 1/10). The concentration of lipids was 1 mg/mL and all the samples were kept at 4 °C overnight and degassed for 10 min before usage. Data acquisitions measured at least 3 scans for lipid DMPC were collected between 5 °C and 45 °C at 10 °C/h, and for lipid DMPS were performed between 10 °C and 60 °C at 30 °C/h. Buffer subtraction and baseline correction were performed using Microcal Origin software (Microcal Inc.).

## 5. Conclusions

In summary, we found that the tryptophan of parent peptide P was an excellent probe to monitor the peptide interacting with different cell membranes and model membranes to illustrate selectivity and anticancer mechanism of action of α-helical peptides. Peptides showed stronger selectivity on cancer membrane mainly due to the charge attraction. Subtle differences on peptide sequence may cause differences of secondary structure, further influencing relative hydrophobicity and self-association ability, then affecting the interaction with target cancer cells. Utilizing tryptophan in peptide sequence as a fluorescence probe appears to be a practical approach to determine the interaction of peptide with phospholipid bilayer membrane. 
